# TRAIL-R1 Is a Negative Regulator of Pro-Inflammatory Responses and Modulates Long-Term Sequelae Resulting from *Chlamydia trachomatis* Infections in Humans

**DOI:** 10.1371/journal.pone.0093939

**Published:** 2014-04-02

**Authors:** Mufadhal Al-Kuhlani, James Rothchild, Sukumar Pal, Luis M. de la Maza, Sander Ouburg, Servaas A. Morré, Deborah Dean, David M. Ojcius

**Affiliations:** 1 Department of Molecular Cell Biology, and Health Sciences Research Institute, University of California Merced, Merced, California, United States of America; 2 Department of Pathology and Laboratory Medicine, University of California Irvine, Irvine, California, United States of America; 3 Laboratory of Immunogenetics, Medical Microbiology and Infection Prevention, Research School V-ICI, VU University Medical Center, Amsterdam, The Netherlands; 4 Institute of Public Health Genomics, Department of Genetics and Cell Biology, Research School GROW, University of Maastricht, Maastricht, The Netherlands; 5 Center for Immunobiology and Vaccine Development, Children's Hospital Oakland Research Institute, Oakland, California, United States of America; 6 Graduate Program in Bioengineering, University of California, Berkeley and San Francisco, California, United States of America; 7 Department of Medicine, University of California San Francisco, San Francisco, California, United States of America; Shanghai Medical College, Fudan University, China

## Abstract

The immune system eliminates *Chlamydia trachomatis* infection through inflammation. However, uncontrolled inflammation can enhance pathology. In mice, TNF-related apoptosis-inducing ligand receptor (TRAIL-R), known for its effects on apoptosis, also regulates inflammation. In humans, the four homologues of TRAIL-R had never been investigated for effects on inflammation. Here, we examined whether TRAIL-R regulates inflammation during chlamydial infection. We examined TRAIL-R1 single nucleotide polymorphisms (SNPs) in an Ecuadorian cohort with and without ***C. trachomatis*** infections. There was a highly significant association for the TRAIL+626 homozygous mutant GG for infection vs no infection in this population. To confirm the results observed in the human population, primary lung fibroblasts and bone marrow-derived macrophages (BMDMs) were isolated from wildtype (WT) and TRAIL-R-deficient mice, and TRAIL-R1 levels in human cervical epithelial cells were depleted by RNA interference. Infection of BMDMs and primary lung fibroblasts with *C. trachomatis* strain L_2_, or the murine pathogen *C. muridarum*, led to higher levels of MIP2 mRNA expression or IL-1β secretion from TRAIL-R-deficient cells than WT cells. Similarly, depletion of TRAIL-R1 expression in human epithelial cells resulted in a higher level of IL-8 mRNA expression and protein secretion during *C. trachomatis* infection. We conclude that human TRAIL-R1 SNPs and murine TRAIL-R modulate the innate immune response against chlamydial infection. This is the first evidence that human TRAIL-R1 is a negative regulator of inflammation and plays a role in modulating *Chlamydia* pathogenesis.

## Introduction

*Chlamydia trachomatis* is the leading cause of bacterial sexually-transmitted diseases (STDs) and the main cause of preventable blindness worldwide _ENREF_1[1]. According to the Centers for Disease Control, there were more than 1.3 million reported cases in the United States in 2010, which corresponds to an increase of ∼8% in comparison to 2008 [2]. The 19 known serovars of *C. trachomatis* are categorized into three disease groups: ocular, urogenital, and the invasive lymphogranuloma venereum (LGV). The latter pathogens include the L_1_, L_2_, L_2a_, and L_3_ strains that infect the reticuloendothelial system involving predominantly the lymph nodes [3], [4].

Infection of epithelial cells by chlamydiae initiates an inflammatory response through ligation of Toll-like receptors (TLRs) and Nod-like receptors [5], [6]. These receptors are usually expressed by immune cells such as macrophages, dendritic cells and neutrophils, but also mucosal epithelial cells [7]–[10]. The engagement of TLRs by microbial products of chlamydiae, such as lipopolysaccharide, initiates the TLR signaling cascade [5], [6]. Once activated, the Toll/IL-1R (TIR) domain of TLR interacts with various adaptors, such as MyD88, which in turn recruits and activates additional adaptor proteins, including the IL-1 receptor-associated kinases (IRAK) and (TNF)-receptor-associated factor 6 (TRAF6) [11]-[13]. TRAF6 then activates various proteins that ultimately lead to the phosphorylation of inhibitor of kappa B alpha (I-κBα), which subsequently undergoes degradation via ubiquitination. The degradation of I-κB releases the activated nuclear factor-κB (NF-κB), which allows it to translocate into the nucleus and stimulate the expression of pro-inflammatory components, such as interleukin (IL)-8, IL-6, IL-18, IL-1α and granulocyte-macrophage colony-stimulating factor (GM-CSF) that recruit and activate various immune cells [14]–[17].

Clearance of infection through inflammation is often an efficient process. However, the mechanisms for clearing chlamydial infection varies among individuals whose immune systems, in addition to clearing the infection, can cause chronic inflammation [18], [19]. Chronic inflammation and tissue damage seen during *C. trachomatis* infections is caused not by the infectious organism, but by the host's immune response to these pathogens. Therefore inflammation needs to be tightly regulated to avoid uncontrolled immune responses.

Negative regulation of inflammation is accomplished at multiple levels throughout the TLR signaling pathways [20], [21]. The first level of regulation involves a decrease in the expression of TLR as the presence of soluble TLRs that can compete with the agonist [22]. Soluble forms of TLR2 and TLR4 dampen the host immune response against infection by preventing the activation of TLR-mediated signaling [23], [24]. Other regulators exert their effect within the cytosol, downstream from TLR ligation. The cytosolic regulators target different components of the TLR signaling pathway such as MyD88, IRAK1, TRAF6, and phosphoinositide 3-kinase [25]–[30].

**Figure 1:**
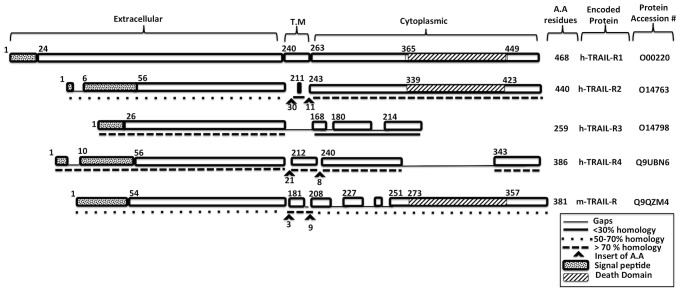
Multiple sequence alignment of human and murine TRAIL-Rs. Sequence alignment shows all four human TRAIL-Rs (hTRAIL-R) and murine TRAIL-R (mTRAIL-R). FASTA files were obtained from the Uniport database at (http://www.uniprot.org). Amino acid (AA) sequences of hTRAIL-R2-4 and mTRAIL-R were aligned in comparison to hTRAIL-R1 using the ClustalW algorithm. The percentage of homology, insertion or loss of AA sequences, and the unique features of the different receptors are presented with different patterns as indicated.

The transmembrane receptor of TNF-related apoptosis-inducing ligand receptor (TRAIL-R) is a member of the tumor necrosis factor receptor superfamily that lacks a TIR domain [31]. In addition to its well-established role in inducing apoptosis, TRAIL-R has been reported to modulate inflammation of the host cells in response to various pathogens and diseases [32]–[34]. Four different TRAIL-Rs have been identified in humans (the transmembrane proteins, TRAIL-R1 through -R4, and a soluble osteoprotegrin) and one full-length receptor in mice [35], [36] (Figure 1). TRAIL-R1 and TRAIL-R2, also known as Death Receptor (DR)-4 and DR-5, are the only known receptors that are capable of selectively killing transformed cells but not normal cells, while TRAIL-R3 and TRAIL-R4 serve as decoys [37], [38]. TRAIL-R-deficient mice develop normal populations of immune cells [33], [39], [40], but challenge of these mice with different pathogens and stimuli for TLR2, TLR3 and TLR4 results in enhanced ability of the innate immune system to clear the infection and increased production of different pro-inflammatory-cytokines such as IFN-β, compared to wildtype mice [33]. The ability of TRAIL-R to downregulate TLR signaling seems to be through decrease activation of NF-κB by stabilizing the I-κBα subunit [33].

While murine TRAIL-R is established as a negative regulator of inflammation, and some progress has been made in understanding the role of TRAIL-R1 in the immune response in humans, nothing is known about the potential role of TRAIL-R1 as a negative regulator of inflammation in humans.

Clear differences in the clinical course of *C. trachomatis* infections in women have been observed, which are not explained by bacterial factors. This suggests that there might be differences in the host genetic background associated with the recognition of pathogens and the immune response that ensues. Indeed, recent research indicates the relevance for studying the host genetic background in relation to disease susceptibility. In one study, an analysis of the lymphoproliferative responses to *C. trachomatis* antigen among Gambian monozygotic versus dizygotic twins in a trachoma endemic region indicated an almost 40% contribution of heritability to the observed differences in the response [41].

The effect of single nucleotide polymorphisms (SNPs) on the function of TRAIL-R1 have not been studied yet in *C. trachomatis* infections. Four TRAIL-R1 SNPs have an effect on the expression of the protein demonstrated as upregulation in silico in human populations based on data in HapMap (http://www.hapmap.org/), but their ability to enhance or diminish inflammation among populations at risk for *C. trachomatis* infection has never been examined.

In this study, we investigate the effect of TRAIL-R1 SNPs in regulating susceptibility to *C. trachomatis* infection among women from a population at risk for STDs, identify TRAIL-R1 as a downregulator of inflammation in human cells, and elucidate the role of murine TRAIL-R in the inflammatory response against chlamydial infection in vitro.

## Results and Discussion

### Association of the TRAIL-R1 SNPs with *C. trachomatis* infection in a human population

A well-defined Ecuadorian cohort with and without ***C. trachomatis*** infections was tested for the four known TRAIL-R1 through -R4 SNPs that could be associated with pathology due to infection.

The human population consisted of 760 women who enrolled in the study. Their ages ranged from 18 to 55 years; the mean age was 29 years. Nine women were excluded from the analyses due to insufficient data. The prevalence of *C. trachomatis* was 16.7% (126 women); there were no patients with human immunodeficiency virus (HIV-1) infection, and 3.2% (4 women) were coinfected with *Neisseria gonorrhoeae*. **Table 1** shows the risk factors for *C. trachomatis* infection. As expected, women younger than 24 years were at increased risk for *C. trachomatis* infection (*p*<0.0003; OR 2.3807; CI 1.4935 to 3.7950), as were women with onset of sexual activity during adolescence. There were no significant differences for infection associated with number of sex partners or prior history of STDs. In addition, the diversity of *ompA* genotypes for the 126 women included Ba (6 women), D (19), E (29), F (25), I (5), J (13), Ja (10), L2 (9), L2b (7), and L3 (3). There were no differences among risk groups for infecting genotype.

**Table 1:** Risk of *C. trachomatis* infection in humans by age group*.

Risk	18–23 yrs (n = 251)		P value	24–29 yrs (n = 250)		P value	>29 yrs (n = 250)		P value
	CT+	CT-		CT+	CT-		CT+	CT-	
Age**	65	186		32	218		27	223	*p*<0.0003
Onset sexual activity:									
adolescence	65	112	P = 0.0018	32	150	*p*<0.018	27	177	*p*<0.0633
adulthood	0	74		0	68		0	46	
No. of sex partners:									
1	27	99	NA	10	75	NA	10	70	NA
>1	38	87		22	143		17	153	
Prior history STDs:									
Yes	40	100	NA	19	111	NA	17	103	NA
No	25	86		13	107		10	120	
Co-STD infections:									
HIV	0	251	NA	0	250	NA	0	250	NA
GC	2	249	NA	1	249	NA	1	249	NA

*Nine women were excluded due to missing data.

**Calculated across all age groups.

The distribution of TRAIL-R1 haplotypes differed between women with and without *C. trachomatis* infection (**Table 2**). The TRAIL-R1+626 homozygous mutant GG was found to be significantly associated with *C. trachomatis* infection versus no infection (*p*<0.0001; OR: 5.3, CI: 2.3763 to 11.8553). There were no synergistic effects for any combination of SNPs. There was no association of any *ompA* genotype with this SNP.

**Table 2:** Distribution of four *TRAILR1* SNPs in *C. trachomatis* positive (CT+) and negative (CT-) women in Ecuador.

	CT+			CT-			P value
	Wildtype	Heterozygote	Mutant	Wildtype	Heterozygote	Mutant	
*TRAILR1* +626 C>G	13.2%	40.4%	46.4%	35.9%	39.9%	24.2%	*p*<0.0001
*TRAILR1* +683 A>C	59.2%	27.1%	13.7%	64.2%	27.9%	7.9%	NS
*TRAILR1* +422 G>A	22.7%	51.9%	25.4%	25.3%	50.9%	23.8%	NS
*TRAILR1* +1387 A>G	83.0%	20.8%	6.2%	79.1%	15.9%	5.0%	NS

We have previously shown the association of host genetic factors (genes encoding TLR-2, TLR-4 and TLR-9) with the susceptibility and course of *C. trachomatis* infection [42]–[44]. Recently, two other studies [45], [46] showed a role for TLR SNPs in the prevalence of pelvic inflammatory disease (PID) among African American women. As TRAIL-R1 can downregulate TLR signaling through decreased activation of NF-κB, our current findings underline the importance of TRAIL-R1 and TLR signaling in the defence against *C. trachomatis* infection. This is consistent with the upregulation of the TRAIL-R1 SNPs in silico.

### MIP-2 expression and IL-1β secretion in TRAIL-R-deficient lung fibroblasts

**Figure 2:**
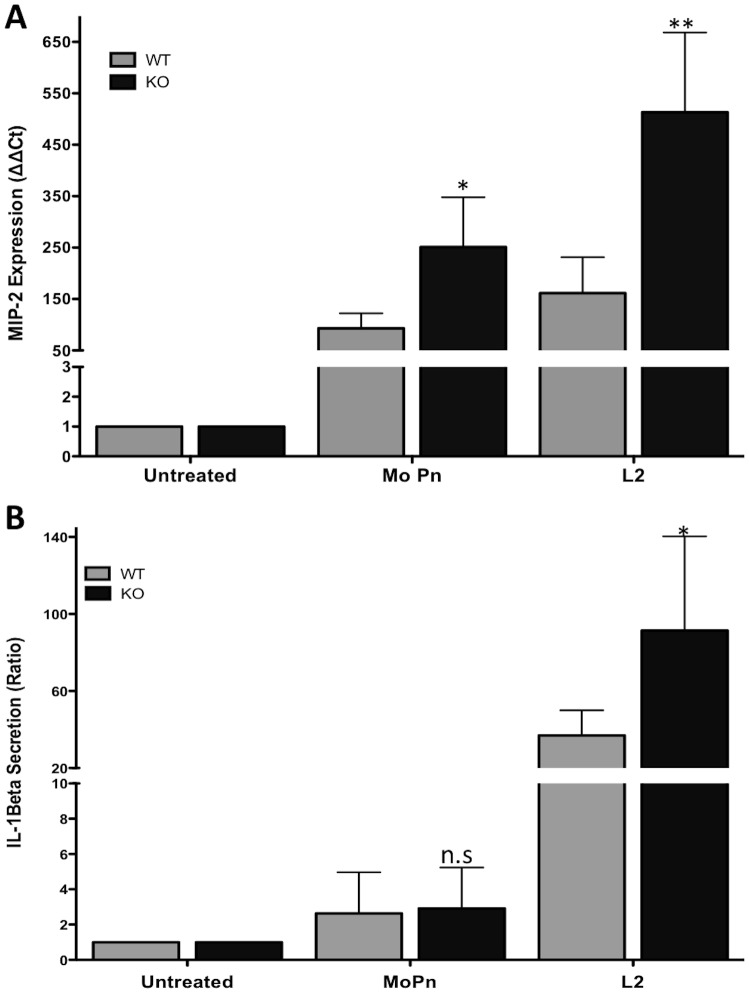
TRAIL-R deficiency increases MIP-2 and IL-1β levels in primary lung fibroblasts in response to a *Chlamydia* infection. WT and TRAIL-R-deficient cells were infected with either *C. muridarum* (MoPn) or *C. trachomatis* (L2) strains at an MOI of 1 for 24 hrs. (**A**) mRNA levels of MIP-2 was measured via qPCR. Cycle threshold (Ct) values were normalized to GAPDH and relative expression (ΔΔCt) was calculated compared to uninfected cells. (**B**) ELISA measurement for IL-1β. The ratio of increased IL-1β level was calculated with respect to uninfected cells. Error bars represent standard deviations from 3 separate experiments. * indicates *p*<0.05; ** indicates *p*<0.01, compared to infected WT cells.

Most previous studies have focused on the ability of TRAIL-R to induce apoptosis, although some studies also report on the effect of TRAIL-R on infection in animal models [47]. A role for TRAIL-R as a downregulator of TLR-mediated signaling has been reported only for the murine TRAIL-R [33]. In this study, the potential role of TRAIL-R in regulating inflammation during chlamydial infection was examined by isolating primary lung fibroblasts from WT and TRAIL-R-deficient mice and infecting them with C. ***muridarum*** **(MoPn) and** *C.* ***trachomatis*** (LGV/L_2_). MoPn is an appropriate strain for infecting lung fibroblasts since it causes pneumonitis in mice. As it has not been possible until now to culture primary cervical epithelial cells from mice, lung fibroblasts were also used to characterize the cellular response to the LGV strain of *C. trachomatis* (L2), which is known to induce a stronger response in the infected cells [48]_,_ [49], [50]. Since *C.* ***muridarum*** is closely related to the *C. trachomatis* human strains, it has been widely used to study the pathogenesis of chlamydial disease in the reproductive and respiratory tracts of the mouse model [49], [50]. As shown in Figure 2A, TRAIL-R-deficient fibroblasts express macrophage inflammatory protein-2 (MIP-2, a murine homologue of human IL-8) at higher levels in response to both *C. muridarum* and *C. trachomatis* infection than WT fibroblasts. However, the secretion of the cytokine IL-1β in response to *C. muridarum* infection was minimal with no significant difference between the WT and TRAIL-R-deficient fibroblasts (Figure 2B). On the other hand, infection of the fibroblasts with the L_2_ strain of *C. trachomatis* induced the secretion of IL-1β more than 60-fold, compared with uninfected cells (Figure 2A). Similarly to the increased MIP-2 expression during *C. trachomatis* infection, TRAIL-R-deficient fibroblasts infected with *C. trachomatis* secrete higher levels of IL-1β than the WT cells (Figure 2B).

Even though MIP-2 expression but not IL-1β secretion is stimulated in response to *C. muridarum* infection, *C. trachomatis* infection led to production of both pro-inflammatory mediators, and their levels were enhanced in the absence of TRAIL-R.

### Expression of pro-inflammatory mediators in TRAIL-R-deficient macrophages

**Figure 3:**
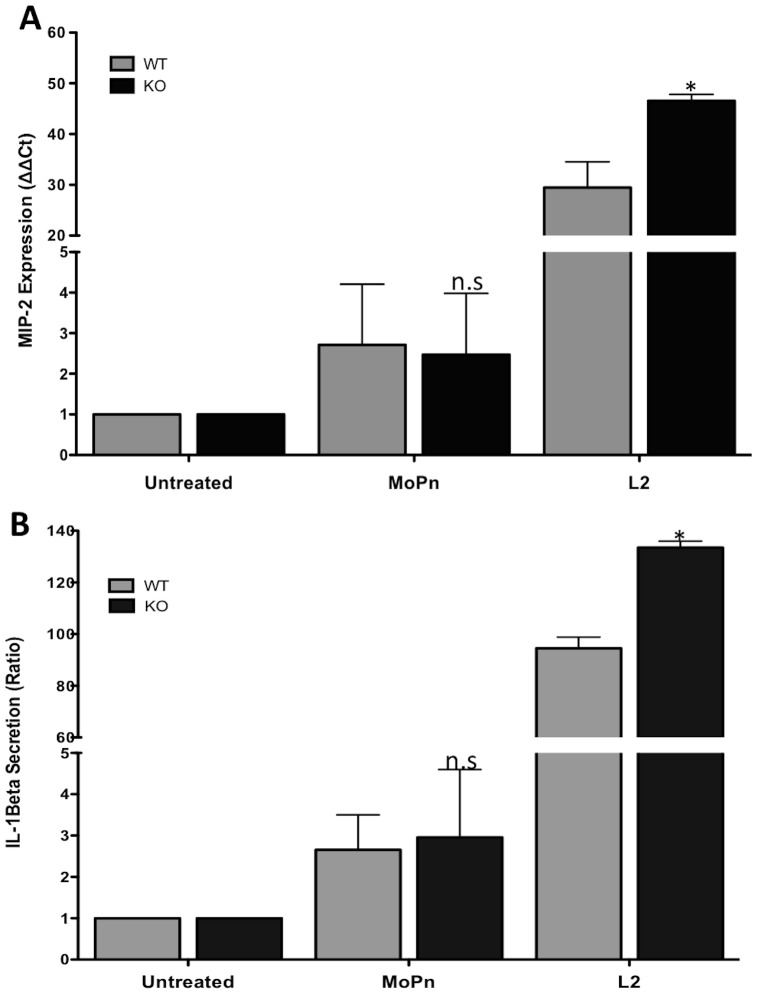
TRAIL-R-deficient BMDMs express higher MIP-2 and IL-1β levels in L2- but not MoPn-infected cells. BMDMs were infected with *C. muridarum* (MoPn) or *C. trachomatis* (L2) as for fibroblasts in Figure 2 above. qPCR analysis was performed for MIP-2 (**A**), and IL-1β secretion was measured by ELISA (**B**). Error bars represent standard deviations from 3 separate experiments. * indicates *p*<0.05; ** indicates *p*<0.01, compared to infected WT cells.

Epithelial cells are the preferred host cells for most chlamydial species, but macrophages can also be infected, or at least stimulated, with *Chlamydia* [51]–[53]. We thus isolated bone marrow cells from WT and TRAIL-R-deficient mice and induced their differentiation into bone-marrow derived macrophages (BMDMs). Flow cytometry analysis, shown in Figure S1, confirmed that the great majority of cultured cells expressed the specific markers, **CD11b** and **F4**/**80**. Compared with the uninfected groups, BMDMs infected with *C. muridarum* expressed low levels of MIP-2 and secreted little IL-1β, with no significant difference between WT and TRAIL-R-deficient cells (Figure 3A, B). In contrast, BMDMs infected with *C. trachomatis* L2 expressed high levels of both MIP-2 and IL-1β, which were enhanced by TRAIL-R deficiency (Figure 3A, B).

### Expression of TRAIL-R1 through TRAIL-R4 in human cells

**Figure 4:**
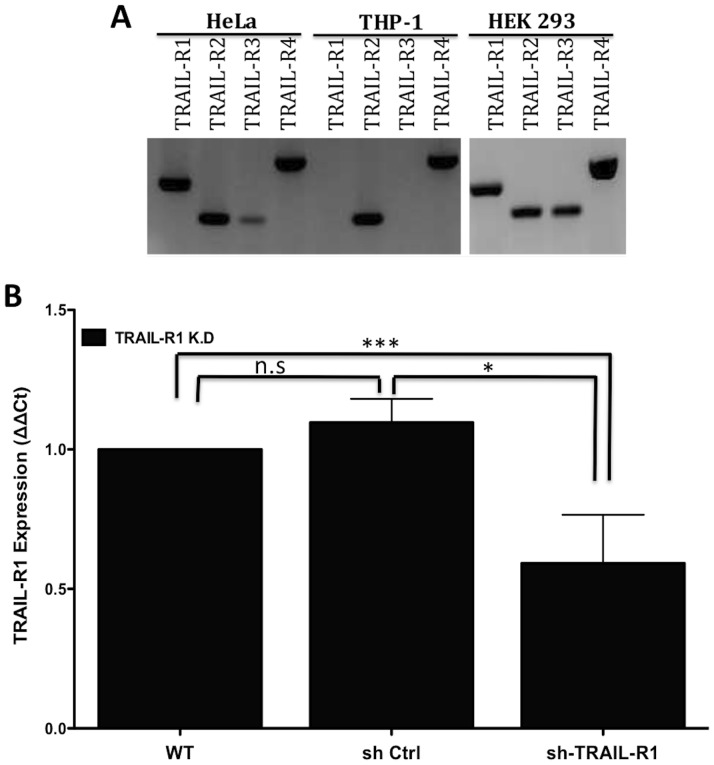
Expression of TRAIL-R1-R4 and depletion of TRAIL-R1 in human cells. (**A**) A 2% agarose gel electrophoresis gel shows RT-PCR amplification of TRAIL-R1 (214 bp), TRAIL-R2 (145 bp), TRAIL-R3 (141 bp) and TRAIL-R4 (271 bp) mRNA from HeLa, THP-1 and HEK293 cell lines. (**B**) HeLa cells transduced with TRAIL-R1 specific shRNA (sh-TRAIL-R1) show a reduced level of TRAIL-R1 mRNA when measured by real-time PCR and compared with wildtype (WT) and non-target control (sh Ctrl). Error bars represent standard deviations from at least 3 independent experiments. *** indicates *p*<0.001 (*p* = 0.0152) and “n.s” indicates “not significant” (*p* = 0.1229) compared with WT. The *p* value for sh-TRAIL-R1 compared to non-target control is 0.0107

With few exceptions, studies of the role of TRAIL-R in regulating inflammation had been performed using the mouse model [33]. We therefore first examined via PCR whether the four human TRAIL-Rs (TRAIL-R1 through TRAIL-R4) are expressed in cells that are commonly used in research on innate immunity and chlamydial infections: the cervical epithelial cell line, HeLa; the human monocyte cell line, THP-1; and the human embryonic kidney cell line, HEK-293. As shown in Figure 4A, both HeLa and HEK293 cells express high levels of all four subtypes of TRAIL-R, with the exception to TRAIL-R3, which is barely expressed in HeLa cells; while the THP-1 cell line expressed only TRAIL-R2 and TRAIL-R4.

### Expression and secretion of IL-8 in TRAIL-R1-deficient cervical epithelial cells

The protein structures of both TRAIL-R1 and TRAIL-R2 resemble each other (Figure 1) [54] [55]. We thus chose to focus our initial studies on TRAIL-R1 given its homology to murine TRAIL-R. To evaluate the role of TRAIL-R1 in vitro, specific shRNA lentiviral particles were used to deplete TRAIL-R1 in HeLa cells. As measured by qPCR, there was a significant reduction of TRIAL-R1 mRNA levels in transduced HeLa cells, compared to the WT and non-target shRNA control cells (sh Ctrl) (Figure 4B).

**Figure 5:**
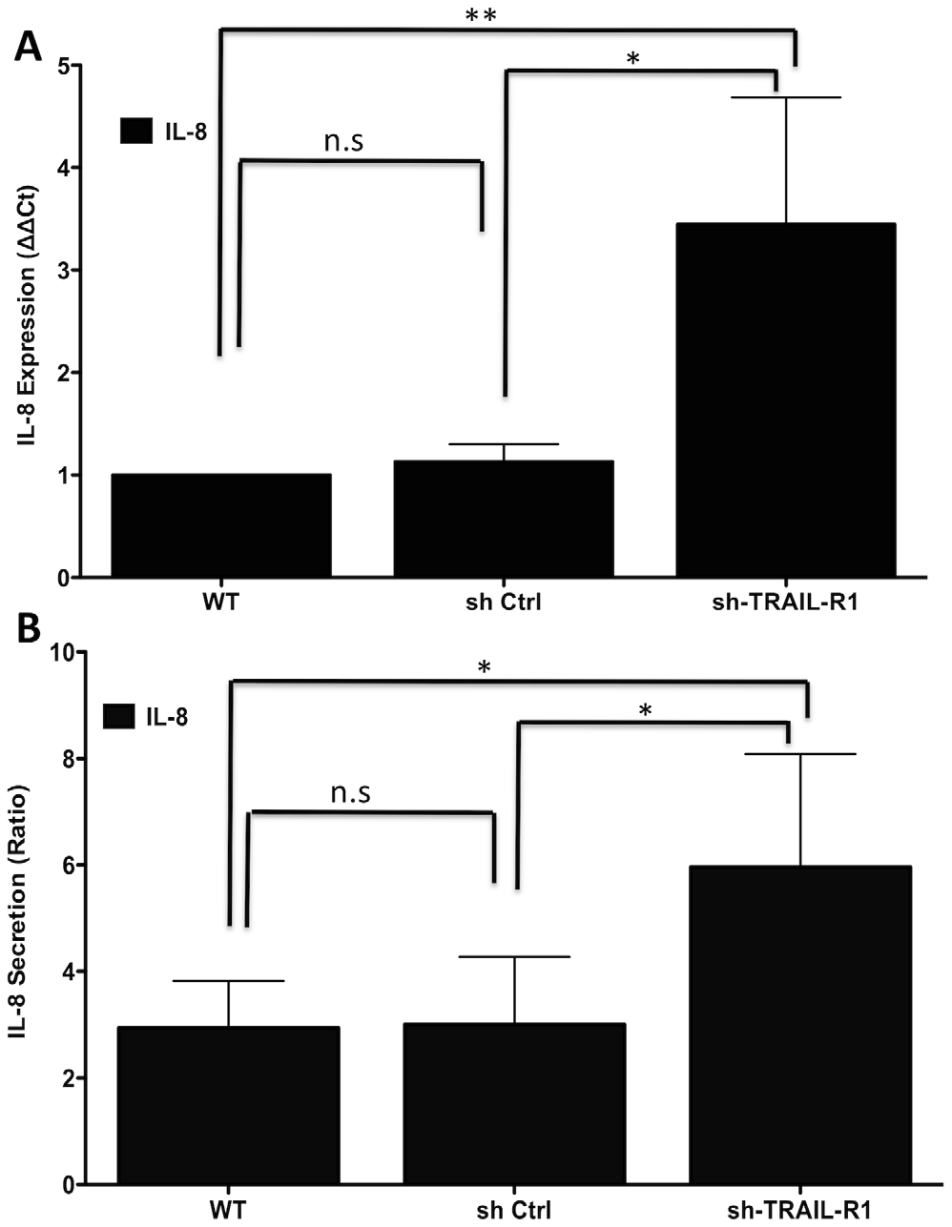
Increased IL-8 expression and secretion in TRAIL-R1-depleted cervical epithelial cells. HeLa cells were infected with *C. trachomatis* strain (L2) at an MOI of 1. (**A**) RNA was isolated 24 hpi for quantification of IL-8 expression using qPCR. The data were normalized to GAPDH, and infected wild type HeLa cells were used to calculate the relative expression (ΔΔCt). Error bars represent standard deviations from 4 separate experiments. ** indicates *p* = 0.0075; and “n.s.” indicates “not significant” (*p* = 0.1736), compared with infected WT cells. The *p* value for sh-TRAIL-R1 compared with non-target control is 0.01. (**B**) ELISA measurement of IL-8 secretion. Error bars represent standard deviations from 4 separate experiments. * indicates *p*<0.05; (*p* = 0.0394); and “n.s.” indicates “not significant” (*p* = 0.9332), compared with infected WT cells. The *p* value for sh-TRAIL-R1 compared to non-target control is 0.0479.

Secretion of the chemokine IL-8, which is an indicator of early stages of the inflammatory response, has been previously described for HeLa cells infected with *Chlamydia* [14], [17], [56]. The levels of IL-8 mRNA expression or IL-8 protein secretion in response to *C. trachomatis* infection in TRAIL-R1-depleted cells were significantly higher than in infected WT or non-target shRNA control cells (Figure 5A, B).

### Concluding remarks

We here show that TRAIL-R can negatively regulate initiation of inflammation in humans and mice during chlamydial infection.

The role of TRAIL-R as a negative regulator of inflammation had been previously addressed in the murine model [33]. The ex-vivo results presented here demonstrate that TRAIL-R is involved in regulation of pro-inflammatory cytokine production in primary lung fibroblasts in response to *C. trachomatis* or *C. muridarum* infection. However, infection with *C. trachomatis*, but not *C. muridarum*, is capable of inducing such responses in the BMDMs. The different responses to *C. muridarum* infection observed between lung fibroblasts and BMDMs, and between *C. trachomatis* and *C. muridarum* infection of BMDMs, suggest that the mechanisms of negative regulation of inflammation by TRAIL-R may depend on the tissues being infected or the specific pathogen strain. In fact, the L2 strain of *C. trachomatis* used in these studies is a pathogen of the reticuloendothelial system involving predominantly the lymph nodes, whereas *C. muridarum* is not.

This is the first report that human TRAIL-R1 also functions as a negative regulator of inflammation. The exact mechanisms used by TRAIL-R1 to dampen pro-inflammatory responses in humans remains to be investigated, but could provide potential biomarkers indicative for the course of infection. The link between TLR signaling and apoptosis has been reported in various studies where proteins that are known to facilitate apoptosis, such as Nur77, PKR or TRAIL, are up-regulated due to activation of some TLR pathways [57]-[62]. Since TRAIL-R1 is a trigger of apoptosis via the adaptor domain, Fas-associated death domain (FADD) and caspase-8, and *C. trachomatis* modulates host cell death during infection, evaluating the effect of TRAILR-R1 on the activation of the apoptotic pathway during chlamydial infection may contribute to explaining the mechanism of action of TRAIL-R1 as a negative regulator. Indeed, the pattern of low mRNA expression of TRAIL-R1 at early times of infection and the increase at later stages (data not shown) could be related to the time-course of *C. trachomatis* infection on apoptosis of the infected cells [34], [63]. Another indicator for the possible involvement of FADD in the function of TRAIL-R as a negative regulator is the observation that FADD can modulate the activity of NF-κB and IL-1β with a pattern that is similar to TRAIL-R [64]-[66]. In addition, the role that the truncated receptors, TRAIL-R3 and TRAIL-R4, may play during inflammation has never been addressed. It is tempting to speculate that, behaving as dominant negative versions of TRAIL-R1, the truncated receptors may have the opposite effect of TRAIL-R1, enhancing rather than dampening inflammation. It is possible that the absence of TRAIL-R results in a decrease of apoptosis in the inflamed tissue and, therefore, increase the intensity of the inflammatory response. Characterization of this gene family may help to gain a better understanding of their role in *Chlamydia* pathogenesis and could lead to the development of therapeutic approaches to minimize the side effects of inflammation when infection occurs.

## Methods and Materials

### Human study populations

Female commercial sex workers (n = 760) ranging in age from 18-55 years were recruited and enrolled in the study through health care clinics in Quito, Ecuador, from August 2002 to August 2005. Potential participants were excluded if they had taken antibiotics within the four weeks preceding study enrollment, had a history of any cancer diagnosis or collagen vascular disease, or were unwilling to complete the study questionnaire and provide biological samples. Written informed consent was obtained from all participants, and approval for the study was granted by the Institutional Review Boards (IRBs) of Children's Hospital Oakland Research Institute and La Pontificia Universidad Católica del Ecuador, Quito, Ecuador, according to the tenets of the Declaration of Helsinki.

All study participants were administered survey questionnaires about risk factors for HIV and other sexually transmitted infections. Participants were excluded from the final analyses if either their *C. trachomatis* diagnostic status or SNP data were missing.

### *C. trachomatis* detection and *ompA* genotyping in patient populations

Cervical swab samples were collected from the study participants using standard techniques and the presence of *C. trachomatis* was determined and commercial nucleic acid amplification tests (NAAT) (Amplicor; Roche Molecular Systems) [67]. Indeterminate samples were resolved using an in-house PCR as previously described [67].

All samples from the Ecuadorian population that were positive by Amplicor and/or in-house PCR were subjected to *ompA* genotyping as described elsewhere [68].

### Haplotype determination

In order to determine the homogeneity or heterogeneity of the Ecuadorian population, the four SNPs for TRAIL-R1 were analyzed. The haplotypes were inferred using PHASE v2.1.1 [69] and SNPHAP [70].

### SNP detection

Genomic DNA was extracted from cryopreserved blood samples using either the DNeasy Blood & Tissue Kit (Qiagen, Valencia, CA) or the MagNaPure LC isolator (Roche Molecular Biochemicals, Germany) according to the manufacturer's instructions. Both techniques provided enough DNA for reproducible genetic analyses. For the Ecuador population, single nucleotide polymorphisms (SNPs) in amplified DNA fragments were identified by the Sequenom MassARRAY system that uses MALDI-TOF Mass Spectrometry. The assays were run at the W.M. Keck Foundation Biotechnology Resource Laboratory at Yale University, New Haven, Connecticut. Custom software was used to design SNP assays and analyze the data. Different size primer extension products were generated for the two alleles of a given SNP. The presence of one or both alleles is scored by the software for each sample. The assays were designed to interrogate four SNPS per sample. These included TRAIL-R1 SNPs +422 G>A (141 His→Arg; rs6557634); +626 C>G (209 Arg>Thr; rs20575); +683 A>C (228 Glu>Ala; rs20576); and +1387 A>G (441 Lys>Arg; rs2230229).

### Statistical analyses

Statistical analyses for the human data were performed as previously described [71]. Briefly, the chi-square test was used for determining frequency of SNP comparisons, the combined effect of the SNPs and risk factors between the groups. Each SNP was tested for Hardy-Weinberg equilibrium. Multivariate logistic regression was used for analysis of STD risk factors, SNPs and SNP interactions. Logic regression was also performed to identify interactions among the SNPs as previously described [71]. Fisher's exact or χ^2^ tests were also used to compare the SNP genotypes between *C. trachomatis* positive and negative STD patients. STATA 10 was used for analyses (Texas). *p* values<0.05 were considered statistically significant.

The statistical significance for the ex vivo and in vitro experiments was evaluated using GraphPad Instat software (GraphPad Software Inc, La Jolla, CA) by unpaired Student's t-test. A value of *p*<0.05 was considered significant. Data are presented as the cumulative result of at least 3 independent experiments, unless stated otherwise.

### Chlamydia stocks

The L_2_ serovar (434 strain) of *C. trachomatis* and *C. muridarum* (previously called *C. trachomatis* mouse pneumonitis (MoPn), strain Nigg II) were grown in HeLa cells as described before [72], [73]. The multiplicity of infection (MOI) for *C. trachomatis* and *C. muridarum* was determined by infecting HeLa cell monolayer cultures as previously described [73].

### Eukaryotic cells

The human cervical carcinoma cell line, HeLa 229, was obtained from American Type Culture Collection (ATCC). The THP1 monocyte cell lines was previously described [74].

To isolate murine cells, TRAIL-R-deficient mice in the C57Bl/6 background were obtained from Dr. Astar Winoto (University of California, Berkeley) [33] and were housed at the University of California, Irvine, Vivarium. The University of California, Irvine, Animal Care and Use Committee approved all animal protocols. The mice were euthanized with an overdose of xylazine plus ketamine. To produce BMDMs, femurs from two WT and TRAIL-R-deficient mice were cleaned from tissues and briefly sterilized in 70% ethanol. BMDMs were prepared as described elsewhere [33]. BMDM cells were harvested using trypsin and the cell populations were analyzed by **flow cytometry** for the expression of BMDM specific receptor using anti-mouse **CD11b-PE** and **F4**/**80-APC** (eBiosciences). Flow cytometry data analysis was performed using FlowJo software, version 7.6.1 (TreeStar).

Primary lung fibroblasts were prepared from wildtype and TRAIL-R-deficient mice, following the procedures previously described [75], [76]. Fourteen days post isolation, the primary lung fibroblasts were seeded into 12 well plates for infection assays, as we previously described [77].

### Generation of TRAIL-R1-depleted HeLa cells using lentiviral shRNA

HeLa cells were seeded in 96-well plates and then transduced in the presence of 8 μg/ml Polybrene for 48 hrs with shRNA-TRAIL-R1 specific lentiviral particles purchased from Sigma-Aldrich (catalog number NM?003842) at a multiplicity of infection (MOI) of 1. Non-target shRNA control cells were also generated using an irrelevant sequence (Sigma, SHC002 V). Cells successfully transduced with lentiviral particles were selected by the addition of media containing 2.5 μg/ml puromycin (Sigma-Aldrich). The knockdown efficiency of TRAIL-R1 was verified by q-PCR analysis.

### Ex vivo and in vitro cell culture and infection

Sixteen to twenty four hours prior to infection, 1×10^6^/well of murine BMDMs, 1×10^6^ murine lung fibroblasts/well, or 5×10^4^ cells/well of HeLa cells were plated on 24 well plates in antibiotic free culture media. Cells growing to about 60% confluency were infected with either ***C. trachomatis*** (LGV/L2) or *C.* ***muridarum*** (MoPn) as indicated at an MOI of 1.0 and incubated at 37°C with 5% CO_2_. Twenty four hours post infection (hpi), supernatants were collected for ELISA analysis and infected cells were lysed in RLT buffer for RNA isolation.

### Reverse transcription and PCR (RT-PCR)

Total RNA were isolated from cells using the Qiagen RNeasy kit (Qiagen) following the manufacturer's instructions. The synthesis of the complementary DNA (cDNA) templates were conducted according to the manufacturer's instructions (TaqMan, Roch). The PCR was performed using Qiagen Fast Cycling PCR Kit. PCR conditions included denaturation steps at 95°C for 5 min, followed by 35 cycles at 96°C for 5 sec, 60°C for 5 sec and 68°C for 5 sec followed by 72°C for 1 min. The PCR products were separated by 2% agarose gel electrophoresis and visualized using ethidium bromide. The primers are listed in Table S1.

### Quantitative-PCR (qPCR)

Total RNA and cDNA were generated as explained above. The q-PCR analysis was conducted in triplicates of 20 μl final volume using Mx3000P (Stratagene, La Jolla, CA) with Brilliant III Ultra Fast STBR Green qPCR master mix (Stratagene). Negative controls include a no-RT control and no cDNA template control (H_2_O alone). Real-time PCR included initial denaturation at 95°C for 3 min, followed by 40 cycles at 95°C for 5 sec, 60°C for 20 sec, and one cycle at 95°C for 1 min, 55°C for 30 sec, and 95°C for 30 sec. The averages of the collected data were normalized to the activity of a house-keeping gene, GAPDH. For mouse BMDM and lung fibroblasts, the relative expressions (ΔΔCt) were calculated using uninfected cells (WT or TRAIL-R deficient) as a baseline. For the human cell line, infected wildtype HeLa cells were used as a baseline for the ΔΔCt calculation.

### ELISA for cytokine secretion

The mouse IL-1β ELISA kit (eBioscience Cat# 88-7013-76) and human CXCL8/IL-8 cytokine ELISA kit (R&D Systems, DY208) were used according to manufacturer's instructions. Ratios of the secreted IL-1β and IL-8 were calculated in comparison to the untreated samples.

## Supporting Information

Figure S1 Representative flow cytometry dot plot of differentiated BMDM cells. Cultured bone marrow cells were stained with BMDM-specific antibodies against mouse CD11b-PE and F4/80-APC, seven days post-isolation.
(TIFF)

Table S1 List of primers used for qPCR.
(TIFF)
