# Correction: TRAIL-R1 Is a Negative Regulator of Pro-Inflammatory Responses and Modulates Long-Term Sequelae Resulting from *Chlamydia trachomatis* Infections in Humans

**DOI:** 10.1371/journal.pone.0106964

**Published:** 2014-08-22

**Authors:** The PLOS ONE Staff

The second author’s name is spelled incorrectly. The correct name is: James Rothschild. The correct citation is: Al-Kuhlani M, Rothschild J, Pal S, de la Maza LM, Ouburg S, et al. (2014) TRAIL-R1 Is a Negative Regulator of Pro-Inflammatory Responses and Modulates Long-Term Sequelae Resulting from *Chlamydia trachomatis* Infections in Humans. PLoS ONE 9(4): e93939. doi:10.1371/journal.pone.0093939

The affiliation for the second author is incorrect. James Rothschild is affiliated with: New York Medical College, New York, United States of America and Center for Immunobiology and Vaccine Development, Children’s Hospital Oakland Research Institute, Oakland, California, United States of America.
