# Supplementary figures and images for: TRAIL-R1 Is a Negative Regulator of Pro-Inflammatory Responses and Modulates Long-Term Sequelae Resulting from Chlamydia trachomatis Infections in Humans
Source: PLoS One. 2014 Apr 2;9(4):e93939. doi: 10.1371/journal.pone.0093939 (PMC3973638; doi:10.1371/journal.pone.0093939)

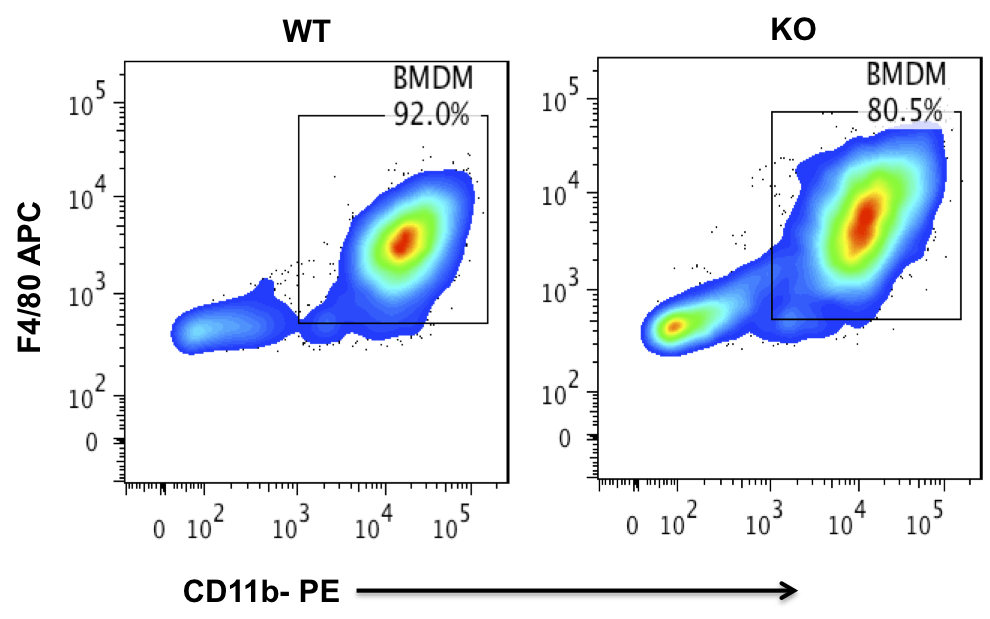

Supplement: Figure S1 — Representative flow cytometry dot plot of differentiated BMDM cells. Cultured bone marrow cells were stained with BMDM-specific antibodies against mouse CD11b-PE and F4/80-APC, seven days post-isolation. (TIFF) [file pone.0093939.s001.tiff]

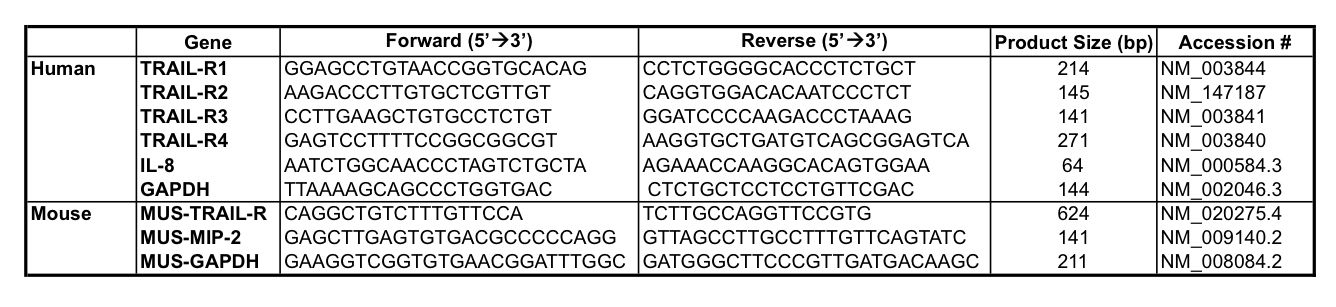

Supplement: Table S1 — List of primers used for qPCR. (TIFF) [file pone.0093939.s002.tiff]
